# The efficacy of tixagevimab/cilgavimab (Evusheld) in prophylaxis and treatment of COVID-19 in immunocompromised patients: a systematic review and meta-analysis

**DOI:** 10.1186/s40001-023-01549-x

**Published:** 2024-01-05

**Authors:** Shaymaa Glhoom, Aya Fergany, Dina El-Araby, Asmaa A. Abdelkhalek, Asmaa Gomaa, Eman O. Zayed, Mohamed Abd-ElGwad

**Affiliations:** 1 Faculty of Pharmacy, Tanta University, Tanta, Egypt; 2 Microbiology and Immunology Department, Faculty of Pharmacy, New Valley University, EL-Kharja, Egypt; 3 Medical Agency for Research and Statistics, Giza, Egypt; 4 Faculty of Pharmacy, MSA University, Giza, Egypt; 5 Zoology Department, Faculty of Science, Al Azhar University, Cairo, Egypt; 6 Faculty of Pharmacy, Cairo University, Cairo, Egypt; 7 Faculty of Medicine, Fayoum University, Fayoum, Egypt

**Keywords:** Cilgavimab, Tixagevimab, Evusheld, AZD7442, COVID-19

## Abstract

**Background:**

During the COVID-19 pandemic, some populations, including immunocompromised patients, could not tolerate COVID-19 vaccination or had low responses. Evusheld is a combined neutralizing monoclonal antibody containing tixagevimab and cilgavimab. The World Health Organization (WHO) has approved this combination as pre-exposure prophylaxis (PrEP) and treatment for immunocompromised patients. With the new variant, the (WHO) recommended an increase in dose from 300 to 600 mg with a booster dose after 6 months. The target of this review was to compare the efficacy of the two doses, 300 mg and 600 mg of tixagevimab/cilgavimab (Evusheld) as prophylaxis for higher-risk individuals to reveal if there is a significant difference in efficacy between those two doses of the drug.

**Methods:**

In this study, electronic databases (PubMed, Web of Science core collection, Scopus, and Cochran) were investigated for articles up to 31/12/2022 in English using a well-established search strategy. We included studies conducted in immunocompromised patients (aged ≥ 12 years) (WHO) received Evusheld as prophylaxis or treatment for COVID-19. After excluding studies inconsistent with the selection criteria, 24 were involved, 22 of which were included in the meta-analysis. We analyzed the data by using RevMan 5.4 program software.

**Results:**

In the double-arm subgroup analysis, Evusheld 600 mg, administered as prophylaxis, showed no significant difference in the COVID-19 infection rate, mortality rate, or needed hospitalization rate compared with the dose of 300 mg (*p* = 0.13, *p* = 0.29, and *p* = 0.25, respectively). In the single-arm subgroup analysis, Evusheld 600 mg, administered as prophylaxis, showed a significant decrease in the COVID-19 infection rate and the hospitalization rate compared with the dose of 300 mg (*p* = 0.0001, *p* = 0.007, respectively). As a treatment, Evusheld showed a significant decrease in the mortality rate over the placebo group (*p* = 0.01) in COVID-19 patients.

**Conclusion:**

This result indicated that Evusheld was an effective prophylactic and therapeutic drug for COVID-19 infection, especially for immunocompromised patients, but there was no considerable variation between the high and low doses. Further prospective and randomized controlled trials (RCTs) with increased population sizes are necessary to show the valuable benefit of the high dose of Evusheld in COVID-19 prevention and treatment and to compare the difference between the two doses within adverse events.

**Supplementary Information:**

The online version contains supplementary material available at 10.1186/s40001-023-01549-x.

## Introduction

The coronavirus disease (COVID-19) pandemic is a significant health issue. Cases of COVID-19 reached approximately 755,703,002 confirmed cases on February 13, 2023, with approximately 6,836,825 deaths [1]. Coronavirus 2, which causes severe acute respiratory syndrome, is the source of COVID-19 (SARS-CoV-2) [2]. In December 2020, the first COVID-19 vaccine was approved by the (WHO) to help control the pandemic [3].

Various COVID-19 vaccinations have been crucial in keeping the pandemic under control. Pfizer-BioNTech and Moderna are two mRNA vaccines that drive cells to produce a non-lethal spike protein that triggers a strong immune response using genetic instructions. Both immunizations demonstrated exceptional efficacy in preventing COVID-19, particularly in severe instances. They contributed significantly to the early vaccination effort [4]. Johnson & Johnson’s Janssen and AstraZeneca-Oxford use viral vector technology, which uses modified adenoviruses to transfer spike protein genetic material. Regarding Johnson & Johnson, this vaccine is administered in a single dosage, which makes it a sensible option for vaccination campaigns. It has been demonstrated that preventing hospitalization and severe sickness is effective. Furthermore, AstraZeneca-Oxford is widely used globally, particularly in lower- and middle-income countries. It has been effective in preventing grave effects [5].

Examples of inactivated vaccines that use killed viral particles to boost the immune system are Sinovac and Sinopharm. These types are dispersed throughout several countries, primarily in Asia and the Middle East. They have effectively prevented hospital stays and serious cases [6]. Novavax uses non-viral components in a protein subunit approach. All of these immunizations have shown to be successful in avoiding COVID-19; some of them are centered around being widely available globally or being simple to administer with a single dose [4].

Vaccines can achieve herd immunity without triggering disease and complications [3]; however, immunocompromised patients (WHO) are more vulnerable to COVID-19 infection with elevated risk for morbidity and mortality and show low postvaccine immune responses [7, 8].

Monoclonal antibodies (mAbs) are considered a new choice that can attain potential therapeutic and prophylactic functions [9]. Combinations of monoclonal antibodies such as casirivimab/imdevimab and tixagevimab/cilgavimab have been used as prophylaxis pre-exposure and therapy [10, 11].

Evusheld was authorized for emergency use as pre-exposure prophylaxis (PrEP) for the prevention of COVID-19 in certain adults and pediatric patients (12 years of age), but after that, on 1/26/2023 (FDA) revised the Emergency Use Authorization (EUA) for Evusheld (tixagevimab co-packaged with cilgavimab). This revision restricts the use of Evusheld to situations where the combined frequency of nonsusceptible SARS-CoV-2 variants nationally is less than or equal to 90% [12].

The prophylaxis of Evusheld has reached a statistically considerable decline in the occurrence of COVID-19 symptomatic infection by 77%. Contrary to the intravenous mAbs that were previously used, this combination is described by two successive intramuscular injections. However, this combination has reported occurrences of allergic reaction reactions with intermittent reports of significant cardiac issues [13, 14].

The suggested dosage is 300 mg, given as successive intramuscular (I.M.) injections of 150 mg each of tixagevimab and 150 mg of cilgavimab. A higher dose of 600 mg (300 mg of tixagevimab and 300 mg of cilgavimab) is suggested to be more suitable for some variants of SARS-CoV-2, especially BA.1 and BA.1.1 [9, 15].

Accordingly, immunotherapy can provide long-term protection to patients with an inadequate COVID-19 vaccination response, leading to a considerable reduction in the risk of COVID-19 infection. Additionally, it has been reported that early treatment with mAbs can diminish hospitalization risk and the rate of mortality within high-risk populations [13, 16].

In previously published meta-analyses and systematic reviews conducted by Alhumaid et al. [17] and Soeroto et al. [18], the safety and efficacy of Evusheld as prophylactic therapy were assessed. Alhumaid et al. demonstrated that Evusheld was effective in significantly reducing the rates of SARS-CoV-2 infection, hospitalization, and mortality compared to control groups. Soeroto et al. similarly found that Evusheld, when used as pre-exposure prophylaxis, significantly reduced hospitalization rates, mortality rates, and SARS-CoV-2 positivity rates compared to control groups. However, neither study evaluated Evusheld as a treatment rather than as prophylaxis. In addition, neither of them compared the safety and efficacy between the two doses Evusheld (300 mg and 600 mg) when applied as prophylactic therapy.

The target of this review is to assess the efficacy of the different doses of 300 mg and 600 mg (Evusheld) as prophylaxis for higher-risk individuals to reveal if there is a significant difference in the efficacy between those two doses. In addition, we aimed to report the clinical outcomes when Evusheld is used to treat immunocompromised patients with COVID-19.

## Method

### Study design

This study assessed the efficacy of Evusheld in the prophylaxis and treatment of COVID-19 in immunocompromised patients. The Selected Reporting Regulation for Systematic Reviews and Meta-Analyses (PRISMA) guidelines [19] and Cochrane Handbook of Systematic Reviews of Intervention [20] were applied to review articles of study. Ethical approval is not essential for assessments of published data. The study was registered on PROSPERO on September 20, 2023, CRD42023465327.

### Eligibility criteria

Using the PICOS framework, our review identified trials that met the following criteria for inclusion. First, the populations were infected with SARS‐CoV‐2 (in prophylaxis research) or with infection (in treatment studies), especially those with an inadequate immune response (e.g., immunocompromised patients, patients with cancer, and organ transplant recipients).

Second, the intervention received Evusheld at any route of administration with any dose during the study period as prophylaxis or treatment for COVID‐19. Third, the control received a placebo or standard of care (SOC) or nothing during the trial. Fourth, the outcomes were reported as the rates of COVID-19 infection (for prophylaxis studies) and other outcomes, such as hospitalization rate, severe symptomatic COVID-19, and mortality rate. The outcomes were reported as COVID-19 infection, hospitalization rate, and mortality rate in prophylaxis studies, but only the mortality rate was assessed in treatment studies. Finally, we included randomized control trials (RCTs), case series, and cohort studies accessible in full‐text form. Studies on pediatrics or pregnant women, abstracts, studies in languages other than English, review articles, comments, and case reports were excluded.

### Literature searching search strategy

We create a well-defined search string using the detected keywords, Boolean operators (AND, OR), and MeSH terms. The medical subject categories included “Evusheld”, “AZD7442”, and “COVID-19” (MeSH). More keywords that were synonyms had been used.

We performed a systematic review by searching different databases (PubMed, Cochrane, Web of Science, Scopus) for articles published in English from January 1, 2020, to January 31, 2023, and subjects related to our search parameters. While reference lists were reviewed, we did not include studies conducted in contexts other than original English-language publications, debates, conference abstracts, or dissertations that were not fully accessible in full.

The first author, Shaymaa Magdy, performed this step. We included all the studies without limitations or filters. This search strategy is described in Additional file 1.

### The process of selection

We performed a systematic review by searching different databases (PubMed, Cochrane, Web of Science, Scopus) for articles published in English from January 1, 2020, to January 31, 2023. We examined the search practice. All documents that resulted from the search strategy were transferred to Endnote software. Then, we removed duplicate articles from the file, and two independent authors (Shaymaa Glhoom and Asmaa A. Abdelkhalek) reviewed the titles and abstracts of the articles for inclusion. Another two independent authors (Eman O. Zayed and Asmaa Gomaa) performed full-text screening by reading the studies carefully to evaluate their suitability. The first author solved any conflicts.

### Data extraction

All the teams participated in this step. The needed data were extracted in organized Excel sheets as follows:

*The general sheet* included the following essential data: the name of the authors, the year of study, the study's design, participants, the aim of the study, main inclusion criteria, intervention, control, the dosage of intervention, and outcome.

*The baseline characteristic sheet* included characteristics of the included studies at the baseline (age, sex, body mass index, and patients with other diseases).

*The outcome sheet* included COVID-19 infection, needed hospitalization, and mortality rate.

We presented continuous data with a quantitative overview of variables like age by utilizing mean and standard deviation measurements. We displayed event and total counts for dichotomous data, giving a descriptive summary of factors like gender. Regarding general characteristics, we gave a narrative summary that included qualitative data such as comorbidities, medical histories, and other relevant information to give a thorough picture of the research population. The parameters were arranged in columns, with each row denoting a distinct study. This arrangement made it possible to compare the parameters between several research projects. Two separate researchers gathered this data and then put it into tables. Lastly, every article chosen by another author was checked to ensure the content was not repeated or overlapped.

### Quality assessment

Using various tools, we assessed the data from the included studies by two independent authors (Dina El-Araby and Asmaa A. Abdelkhalek). First, the Newcastle–Ottawa Scale (NOS) [21] was utilized to evaluate the risk of bias in cohort studies. This tool assessed the quality of observational studies on three essential domains: subject selection, the equivalence of individuals about demographics and critical potential confounders, and the ascertainment of the predetermined outcome. The final collective score that each study could obtain ranged from 0 to 9, where a score ≥ 7 was classified as a good-quality trial.

*Second*, the tool used to estimate the risk of bias in case series studies was based on nine criteria [22]. Those criteria include the study question or objective specified, the population of the study and thoroughly described the cases consecutively, the subjects comparable, the intervention the outcome measures highly defined, the duration of follow-up adequate, and statistical methods and the results thoroughly explained. If the Quality Assessment Tool for Case Series Studies fulfilled eight criteria, it showed good quality, five criteria revealed reasonable quality, and three criteria indicated poor quality.

Third, the revised Cochrane risk-of-bias tool version 2 (RoB 2.0) [23] was used to evaluate RCTs based on five assessment domains: bias occurring from the process of randomization, bias due to differences from intended interventions, bias due to missed outcome data, bias in measuring the outcome, and bias in the choice of the stated result. The authors’ evaluation is classified as low risk, high risk, or particular concerns of bias.

### Statistical analysis

We utilized Review Manager (RevMan) software version 5.4.1 for statistical analysis of double-arm studies. Meta Analyst software was used for single-arm analysis. The pooled risk ratio (R.R.) was utilized for dichotomous data, and the mean difference was applied to continuous data. For analysis, we employed the random-effects model. *p* values < 0.05 were considered significant. A leave-one-out test or subgrouping analysis was adopted to solve heterogeneity [24].

### The assessment of heterogeneity

We assessed heterogeneity by the *I*-square (*I*^2^) test [25] and the *p*-value. Heterogeneity degrees were classified based on *I*^2^ values (not significant: 0–40%; moderate: 30–60%; substantial: 60–80%; and significant: 80–100%). Analysis was deemed heterogeneous if the *p*-value was lower than 0.05 or the *I*^2^ was higher than 60%. A leave-one-out test [26] or subgrouping analysis was used to address heterogeneity.

## Results

### Selection and characteristics

**Fig. 1:**
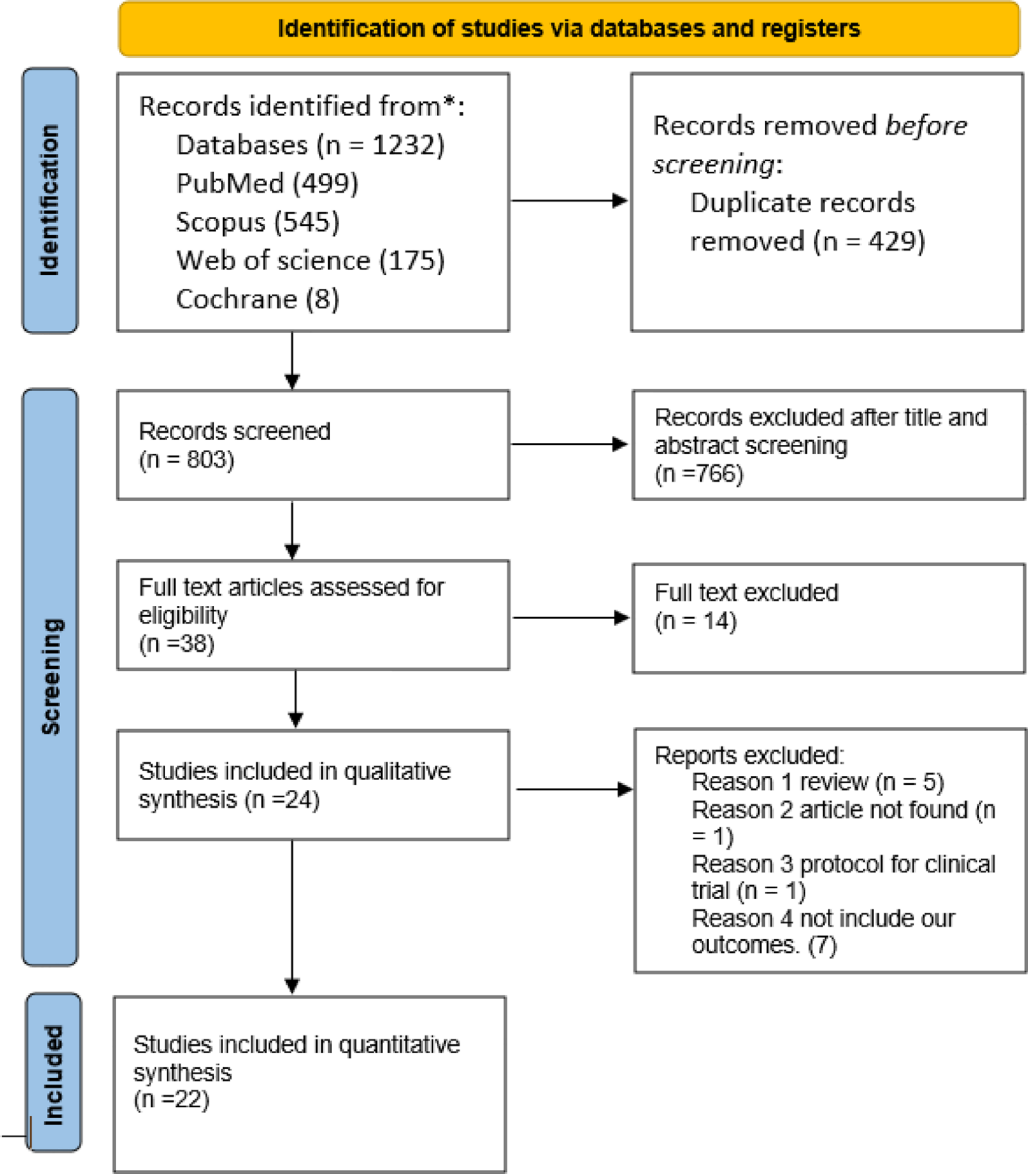
A PRISMA diagram shows the included studies

After searching databases, including PubMed, Web of Science, the Cochrane Library, and Scopus, we obtained 1219 records. After removing 429 duplicates, the abstract of the remaining 790 studies were examined for the inclusion criteria. Twenty-four whole papers were examined for appropriateness. A comprehensive overview of the 24 included trials is clarified in Table 1. Of them, two articles were further excluded because of low sample sizes. Finally, 22 studies [8, 22–42] were included in the meta-analysis (Fig. 1, PRISMA flowchart).

**Table 1:** This table shows a comprehensive overview of the included trials

Author, year, and country	Study design	Participants and primary inclusion criteria	Age		Gender/male	Intervention	Control	Dosage of intervention	Outcome
Benotmane et al. 2022 [38] France	Retrospective cohort study, 28, 2021 to March 13, 2022, in France	KTR patients who had previously received the combination of casirivimab-imdevimab may also have been exposed to those who had sub-optimal responses to SARS-CoV2 mRNA vaccinations	Median (IQR) 60.1 (52.3–71.9)		23/39 (59)	I.M Evusheld	Null	150 mg tixagevimab and 150 mg cilgavimab	For SARS-CoV-2 IgG II Quant A testing, a titer of 7.1 BAU/mL (50 arbitrary units/mL) or more significant for anti-RBD IgG antibodies was determined as the positive limit. The results of a 1:40 dilution of sera were considered positive for neutralizing antibodies if they neutralized 50% or more of the SARS-CoV-2 pseudovirus
Kertes et al. 2022 [47] Israel	Retrospective observational study	Age 12 and above, 40 kg or more, no recent positive test results for COVID-19 (PCR or antigen), no recent COVID-19 vaccination, and significant immunosuppression, as defined by the International Medical Organization for Health (IMOH)	12 to 39 40 to 59 60 to 69 70 to 79 80+	4.1 29.9 28.6 30.5 6.8	512 (62.1)	I.M AZD7442	Did not receive AZD7442	300 mg: 150 mg tixagevimab and 150mg 5 cilgavimab	SARS-CoV-2 infection was the primary endpoint of the study, determined by the presence of 14 positive polymerase chain reactions (PCR) or antigen test results over the follow-up period. The trial's secondary endpoint was severe COVID-19 illness, measured in every group during simultaneous periods and determined as COVID-19-related hospitalization and all-cause fatalities
Nguyen et al, 2022 [27] France	An observational multicentre cohort study in nine divisions on three University Hospitals found in Ile-de-France between December 28, 2021, and March 31, 2022	Patients with impaired immunity who get tixagevimab/cilgavimab as pre-exposure prophylaxis after at least three doses of vaccination had a Reduced response, as measured by anti-spike IgG and antibodies	Mean (SD) 58.9 (20.7)		Not reported	Tixagevimab/cilgavimab as pre-exposure prophylaxis	None	Tixagevimab/cilgavimab 150/150 mg intramuscularly	This study aimed to report the occurrence and outcomes among immunosuppressed patients who received Evusheld as prophylaxis in France. The omicron wave, where positive re-time PCR of SARS CoV-2. confirmed COVID-19 infections
Thomas et al. 2022 [40] ACTIV-3–(TICO) Study Group Multicounty	Randomized controlled trial	18-year-old patients with COVID-19 symptoms spent up to 12 days in the hospital	55 (44–66)		1579	Tixagevimab/cilgavimab	Placebo	Tixagevimab 300 mg/cilgavimab 300 mg	The primary outcome was the time to prolonged recovery through day 90. The secondary outcomes included mortality at 90 days and a combined safety result (death, severe adverse events, organ failure, and serious co-infection)
Young et al. 2022 [31] United States	Retrospective cohort study	Veterans older than eighteen. Patients in the treatment arm got at least one dose of tixagevimab/cilgavimab at some point during the observation period. Those patients who did not get tixagevimab/cilgavimab but were nonetheless considered high-risk were included as controls	Mean (S.D.) 67.4 (11)		1579 (91)	Intramuscular tixagevimab/cilgavimab	Immunocompromised or other high-risk patients who did not receive tixagevimab/cilgavimab	(150 mg/150 mg) then increased to 300 mg /300mg	SARS-CoV-2 infections were approved using the reverse transcriptase polymerase chain reaction (RT-PCR) or antigen testing. A positive SARS-CoV-2 RT-PCR result or antigen test within 30 days of a hospital admission or discharge diagnosis for COVID-19 was considered hospitalized for the virus. A date of death (DoD) during follow-up was deemed all-cause mortality
Levin et al. 2022 [11] Multicounty (PROVENT)	The parallel-group, randomized, double-blind, multicenter, placebo-controlled trial, The primary analysis data cut-off date was May 5, 2021. The primary objective, significant supporting analyses, and significant secondary endpoints all had an additional prolonged follow-up data deadline on August 29, 2021. The trial is anticipated to end on June 29, 2022	A screening SARS-CoV-2 serologic test result that was negative for all subjects was required. Individuals with a history of COVID infection were excluded, and those who were elderly (60 years or older) and obese were more likely to be immunocompromised, unable to receive the COVID-19 vaccination without experiencing negative side effects, or to have chronic kidney disease, heart problems, chronic obstructive respiratory disease, or chronic liver disease. Also, the study involved participants with an increasing probability of catching SARS-CoV-2	Mean (S.D.) 53.6 (15)		1865 (53.9%)	Evusheld	Placebo	Evusheld	The beginning of symptomatic COVID-19, as determined by confirmed findings from RT-PCR testing and started after the administration of Evusheld or placebo and on or before 183 days, was the primary efficacy endpoint
Lafont et al. 2022 [28] France	A monocentric observational retrospective study on December 20, 2021, and March 15, 2022	15 individuals in the prophylactic group and 13 in the treatment group, including immunocompromised patients older than 18 years with COVID-19-positive PCR or antigen test	Median (IQR) 56 (44–63)		8 (53)	Tixagevimab/cilgavimab	No	300 mg	The death rate was significantly lower in the treated group when patients who received targeted anti-SARS-CoV-2 therapy with no prophylaxis and those who did not were compared. In patients getting treatment, there were no grave adverse effects noted. Six out of fifteen patients who received tixagevimab/cilgavimab as pre-exposure prophylaxis had later curative therapy, and there were no deaths associated with COVID-19
Ordaya et al. 2022 [30] United States	Descriptive analysis	All patients who received the first allowed dosage of tixagevimab 150 mg co-formulated with cilgavimab 150 mg and were 18 years or older were included in this research	Median (IQR) 57 (28.7–71.5)		2 (25)	Tixagevimab 150 mg co-formulated with cilgavimab 150 mg	No	Tixagevimab 150 mg co-formulated with cilgavimab 150 mg	Patients who acquired COVID-19 after 2 months of Evusheld exposure
Levin et al. 2022 (storm chaser) [42] United States	Randomized controlled trials (RCT)	Individuals were ≥ 18 years old with possible exposure to an infected covid person with or without symptoms within eight days. Participants should not have had symptoms of COVID-19 within ten days	Mean (S.D.) 46.6 (15.7)		376 (50)	AZD7442	Placebo	300 mg AZD7442	The primary safety outcome was the frequency of adverse events, and the primary efficacy outcome was the occurrence of symptomatic COVID-19 after drug or placebo administration
Montgomery 2022 [39] (TACKLE) Multicounty	Between January 28, 2021, and July 22, 2021, a randomized, double-blind, controlled study	Eligible participants were persons aged 18 years and older who were not currently hospitalized and whose SARS-CoV-2 infection had been confirmed by a laboratory using RT-PCR (or an antigen test on a respiratory sample taken within the last three days	46·3		213 (47%)	Evusheld	Placebo	600 mg dose, one each of 300 mg tixagevimab and 300 mg cilgavimab)	A combination of severe COVID-19 or death through day 29 served as the primary effectiveness outcomes; pneumonia or hypoxemia were considered indicators of severe COVID-19. Furthermore, respiratory failure protection
Karaba et al. 2022 [46] The United States	A prospective observational cohort study	61 Solid organ transplant patients who were vaccinated	62.5 (57.7–68.5)		25 (41)	Evusheld		600 mg I.M	The binding antibody and neutralization surrogate were evaluated against Omicron siblings BA.1 and BA.2 variants
Jondreville et.al. 2022 [41] France	Retrospective multicentric study	Allo-HSCT patients who used Evusheld as prophylaxis	Median (IQR) 58 (21–74)		Not reported	Evusheld as prophylaxis	No	300-mg Evusheld (tixagevimab and cilgavimab, A 150 mg each)	The frequency and seriousness of SARS-CoV-2 infections in allo-HSCT recipients who obtained Evusheld for prophylaxis
Davis et al. 2022 [35] The United States	Retrospectively analyzed medical records from January 2022 to August 2022	People who received Evusheld as prophylaxis and had B cell cancer	Median (IQR) 66 (18–91)		149 (59)	Evusheld as prophylaxis	No	Both 300, 600, and 900 mg dose	There were no fatalities and only a few people with infections needed to be hospitalized
Calabrese et al. 2022 [37] The United States	Retrospective cohort—deidentified	Patients with BCDT or humoral IEI who received at least one dose of tixagevimab/cilgavimab and were subsequently diagnosed with COVID-19 were included	Median, 64		4 (33.3)	Tixagevimab/cilgavimab prophylaxis		300 mg I.M. 6 patients 600 mg I.M. 6 patients	Eleven persons had a slight illness and recovered at home. One person who was hospitalized required high-flow oxygen. There was no death
Aqeel et al. 2022 [48] The United States	Retrospective cohort study between December 2021 and June 2022	Twenty-one patients with antibody-associated vasculitis (AAV) received rituximab for remission induction and maintenance. They also received Evusheld at a dose of 600 mg	Mean (S.D.) 66 (15.5)		Not reported	Evusheld prophylaxis	None	600 mg (300 mg tixagevimab-300 mg cilgavimab)	The outcomes included CD19, IgG, and the progress of COVID-19 infections before and after treatment with Evusheld
Totschnig et al. 2022 [33] Austria	The retrospective study	Pre-exposure prophylaxis with TIX/CIL was administered to 37 immunocompromised outpatients, and subsequent infections with SARS-CoV2 were monitored	Mean (S.D.) 59.6 (15.1)		53 (59.5)	Evusheld Prophylaxis	None	TIX/CIL 150 mg/150 mg '(IM)'	Tracking the recurrence of infections and tracking the development of antibodies
Al Jurdi et al. 2022 [43] The United States	A retrospective multicenter cohort study	222 (SOTRs) who received Evusheld as prophylaxis	Median (IQR) 65 (55–72)		136 (61.3)	Tixagevimab/cilgavimab prophylaxis	None	150–150 mg dose 300–300 mg dose	
Goulenok et al. 2022 [49] France	Retrospective, case series; single center	HIV/AIDS patients with IMIDs (AIDs and systemic vasculitis) Ten patients with severe immunosuppression were given TGM/CGM (intramuscular) (150–150 mg dose) Seven individuals with a severe immune deficiency were not given any mAbs	Median (IQR) 52 (19–75)		4 (40)			150–150 mg dose	Two of t the patients who tested positive for SARS-CoV-2 by RT-PCR developed COVID-19 (both were Omicron) Two-thirds of those exposed to TGM/CGM exhibited symptoms of COVID-19 Two-thirds of those who contracted COVID-19 had mild cases after taking TGM and CGM together Hospitalization due to COVID-19 was necessary for 2/3 of those infected
Kaminski et al. 2022 [45] France	Retrospective cohort; single center	333 KTRs were equipped with TGM/CGM (I.M.) 97 KTRs were given a placebo	Mean (S.D.) 60 (14.4)		204 (61.2)		Placebo	150–150 mg dose	COVID-19 hospitalization was significantly reduced in the TGM/CGM group, with a significant reduction in ICU admissions. Only one patient in the TGM/CGM group died, whereas two in the placebo group
Debbiny et al. 2022 [34] Israel	Retrospective cohort; multicenter	703 immunocompromised patients received TGM/CGM (I.M.)	Mean (S.D.) 66.2 (13.7)		402 (57.2)			150–150 mg dose	The TGM/CGM group had lower SARS coronavirus type 2 RT-PCR positivity than the non-TGM/CGM group. In the COVID-19-linked rate of hospitalizations, its frequency was decreased on the TGM/CGM side than the other side
Ollila et al. 2022 [29] United States	Retrospective cohort; single center	25 hematologic malignancy patients received TGM/CGM (I.M.)	Not reported		Not reported		12 hematologic malignancies patients received no treatment	150–150 mg dose	SARS coronavirus two reverse transcription polymerase chain reaction positivity: the TGM/CGM group had fewer SARS-CoV-2 positive PCR results (zero vs three, *p* = 0.007). Only one patient who did not receive any treatment died
Zerbit et al. 2022 [32] France	Prospective cohort; single center	57 individuals with hematologic malignancies were given TGM/CGM (intramuscular) (150–150 mg dose)	Median (IQR) 71 (63–78)		36/57 (63)		No TGM or CGM was given to 236 individuals with hematologic malignancies	150–150 mg dose	Reverse transcription polymerase chain reaction positivity for SARS-CoV-2 was lower in the drug group than in no drug one (5/102 vs. 52/236, p 0.05)
Bertrand et al. 2022 [44] France	Retrospective cohort; single center	412 KTRs patients	Mean (SD) 60.2 (14.2)		254 (61.6)		62 KTRs received CRM/IDM (IV)(600–600 mg dose)and 98 KTRs received.no mAbs		COVID-19 infection rate was decreased in the drug group than in no drug one (28 vs. 56, p 0.001) Hospitalization due to COVID-19: the proportion of patients hospitalized was decreased in the drug group than in no drug one Also, patients in the drug group needed fewer ICU admissions Patients assigned to TGM/CGM had no deaths, but five were CRM/IDM side or no mAbs and died
Cochran et al. 2022 [36] United States	Retrospective cohort; single center	TGM/CGM (I.M.) was administered to 205 individuals with SOTR; the dosage ranged from 150 to 150 mg (*n* = 14) and 300 to 300 mg (*n* = 191)	Not reported		Not reported				Patients who got the lower dosage of TGM/CGM (150–150 mg) had a higher possibility of possible positive covid 19 than those who took the higher dose of TGM/CGM (300–300 mg) (4/14 vs 12/156) One person in fourteen receiving the lower dosage of TGM/CGM (150/150 mg) was hospitalized because of COVID-19, whereas two people in 156 receiving the higher dose (300/300 mg) were admitted. Deaths from COVID-19 were more common in those receiving the lower dosage of TGM/CGM (150/150 mg) than those receiving the higher dose (300/300 mg)

Of the 24 included studies, 17 were observational cohort studies, four were double-blinded RCTs, and two were case series studies. All trials involved participants with inadequate immune responses to SARS-CoV-2 immunization or immunosuppressed persons, such as cancer patients and recipients of solid organ transplants. Most included trials injected 150 mg of tixagevimab and 150 mg of cilgavimab intramuscularly (I.M.). Seven studies used a formulation of 300 mg/300 mg. Some additional outcomes are not included in our meta-analysis but are written in Table 2.

**Table 2:** This table shows additional outcomes for some studies

Author and year	Significant adverse events	Severe illness symptoms	Mild illness symptoms	Sustained recovery	Patients entered ICU	Oxygen requirements
1. Benotmane et al. 2022 [38]					Only 3 of 416 patients had entered ICU	
2. Kaminski et al. 2022 [45]	–	Within (333), (2) COVID-19-related hospitalization in the intensive care unit			Only 2 of 333 patients had entered the ICU in the intervention group; in the control group, six from 7 had entered the ICU	
3. Nguyen et al. 2022 [27]		–	–			6 of 49 patients needed oxygen
4. Lafont et al. 2022 (for prophylaxis) [28]		–			1 of 15 had entered the intervention group, but two of 10 in the control group	2 of 15 patients needed oxygen in the intervention group. However, in the control group, three out of 10 needed oxygen
5. Lafont et al. 2022 (for treatment) [28]	Not reported	Within a total of l 42, (5) reported oxygen requirements, (12) hospitalization, (2) intensive care	Within a total of l 42, (2) reported asymptomatic, (26) fever, (28) cough, (15) dyspnea,		2 of 13 had entered the ICU in the intervention group, but two of 10 in the control group	2 of 13 patients needed oxygen in the intervention group, but in the control group, there were three from 10
6. Ordaya et al., 2022 [30]	Only two individuals had severe respiratory symptoms, while other patients showed mild respiratory symptoms with no mortality	Within eight patients: (2) showed bacterial complications, and (1) needed oxygen therapy	Ranging from asymptomatic to cough, dyspnea,			Only one patient out of 8 needed oxygen
7. Levin et al., 2022 (PROVENT) [11]	Total 3461 include (64) sever, (50) serious, and (93) particular interest adverse events, including (82) Injection-site reactions	–				
9. Thomas L et al.,2022 [38] ACTIV-3-(TICO) [37]	–			Of 710 patients, 17 sustained recovery in the intervention group, but 595 from 707 sustained recovery in the control		
10. Karaba et al. 2022 [46]	Through 7 days of any T+C injection, no subjects stated any adverse cardiac events, hypersensitivity reactions, or acute organ rejection (reactions that were reported were mild to moderate)					
11. Jondreville et al. 2022 [41]	Only one patient of, 161 had significant adverse events					
12. Calabrese et al. 2022 [37]	Only one of 412 patients had significant adverse events		11 of 412 patients had mild symptoms			
13. Goulenok et al. 2022 [49]		None from 10 patients had a severe illness in the intervention group, but in the control group, four of 7 patients had severe illness symptoms				
14. Bertrand et al. 2022 [44]		Only 2 of 412 patients had severe illness symptoms in the intervention group. However, in the control group, 6 160 had severe illness symptoms			In the intervention group, 2 of 412 entered the ICU, but in the control group, entered the ICU	

### The risk of bias in studies

**Fig. 2:**
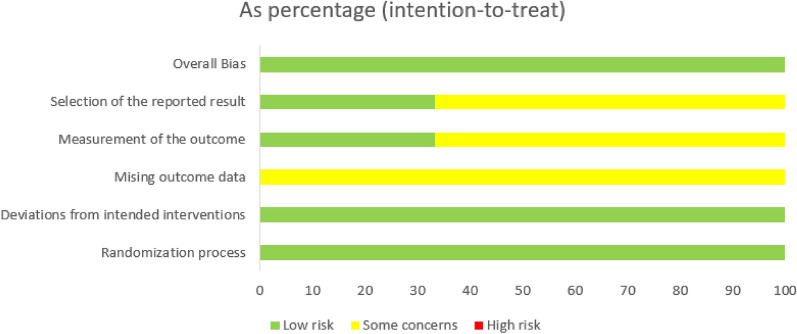
This figure summarizes the quality assessment of the three included studies

**Fig. 3:**
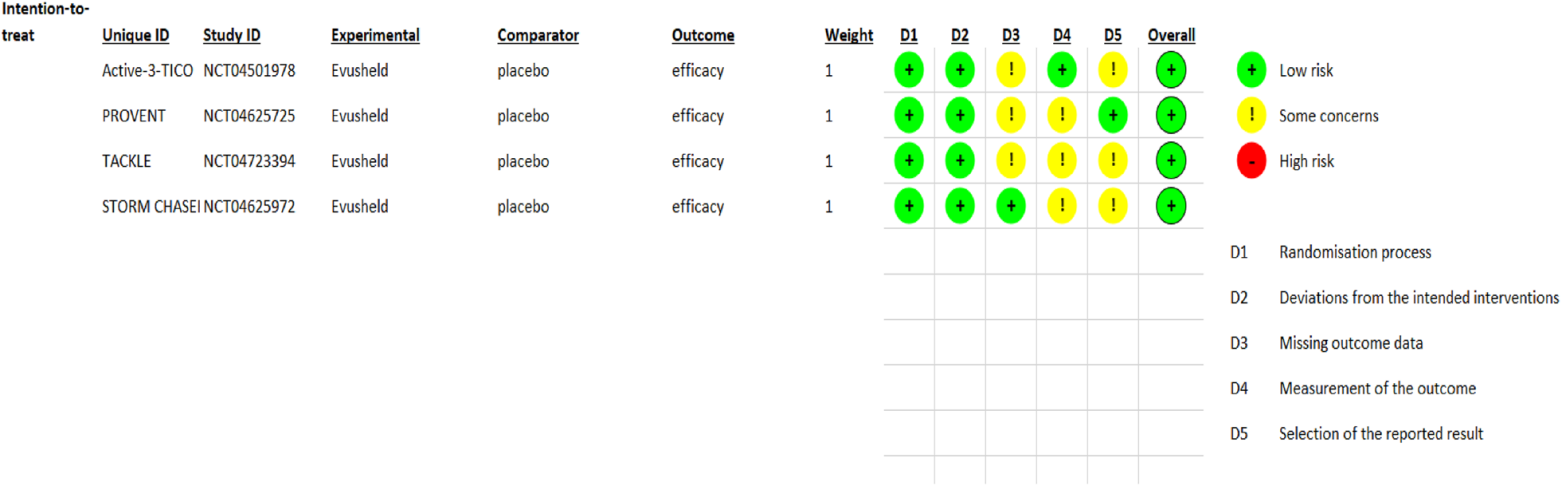
The Risk of Bias version 2 (Rob v2) graph for assessment of randomized controlled trials

Among 17 assessed cohort studies, 11 research were of good quality (i.e., NOS scores were above 7) (Additional file 2: Table S1). The four randomized, double-blind controlled trials had a low risk of bias regarding RoB (Figs. 2 and 3). Three case series studies showed quite good quality (i.e., scores > 7 based on the assessment of bias by Quality Assessment Tool for Case Series Studies) (Additional file 2: Table S2).

1. A point was given if the exposure data came from a prescription registry or a medical file.
2. A point was given if the study was prospective in design.
3. If age adjustments were made, a point was given.
4. If medicines (such as anti-hypertensives, anti-diabetics, etc.), a point was given.
5. A point was given if the follow-up was completed with 80% accuracy or more.

According to Risk of Bias version 2 (RoB v2), the summary of the four included studies’ quality assessments showed a 100% low risk of overall bias. Also, the randomization process domain and deviation from intended interventions domain were 100% low risk of bias. However, missing outcome data domain was 100% some concerns of bias, while the two domains, measurement of the outcome and selection or the reported result, were only 30% low risk of bias (Fig. 2).

Regarding each study of the four randomized control included trails, all four studies were low risk of bias. Each study had at least three domains with low risk of bias and two domains with some concerns of bias. Additionally, there was no domains with high risk of bias (Fig. 3).

### Publication bias

It was not applied in this review because it had less than ten studies in each outcome.

### Clinical outcomes

#### The analysis for prophylaxis

##### COVID-19 infection

Double-arm subgroup analysis of COVID-19 infections by comparing Evusheld dose groups 300mg and 600mg included twelve studies. The 600 mg dose group included three studies with 2276 patients compared to 6736 patients in the control group. The 300 mg dose group included nine studies with 6681 patients compared to 9941 patients in the control group.

The results revealed no substantial variation in COVID-19 infections between the two compared dose groups (*p* = 0.13). However, there was a highly significant decrease in the number of COVID-19 infections favored by the Evusheld 600 mg dose group over the other group (*p* < 0.00001). Also, there was a highly significant decrease in the number of COVID-19 infections favoring the Evusheld 300 mg dose group over control individuals (*p* < 0.000). There was a highly significant decrease in the number of COVID-19 infections favoring Evusheld groups (300 mg and 600 mg doses) than the control groups (*p* < 0.00001).

**Fig. 4:**
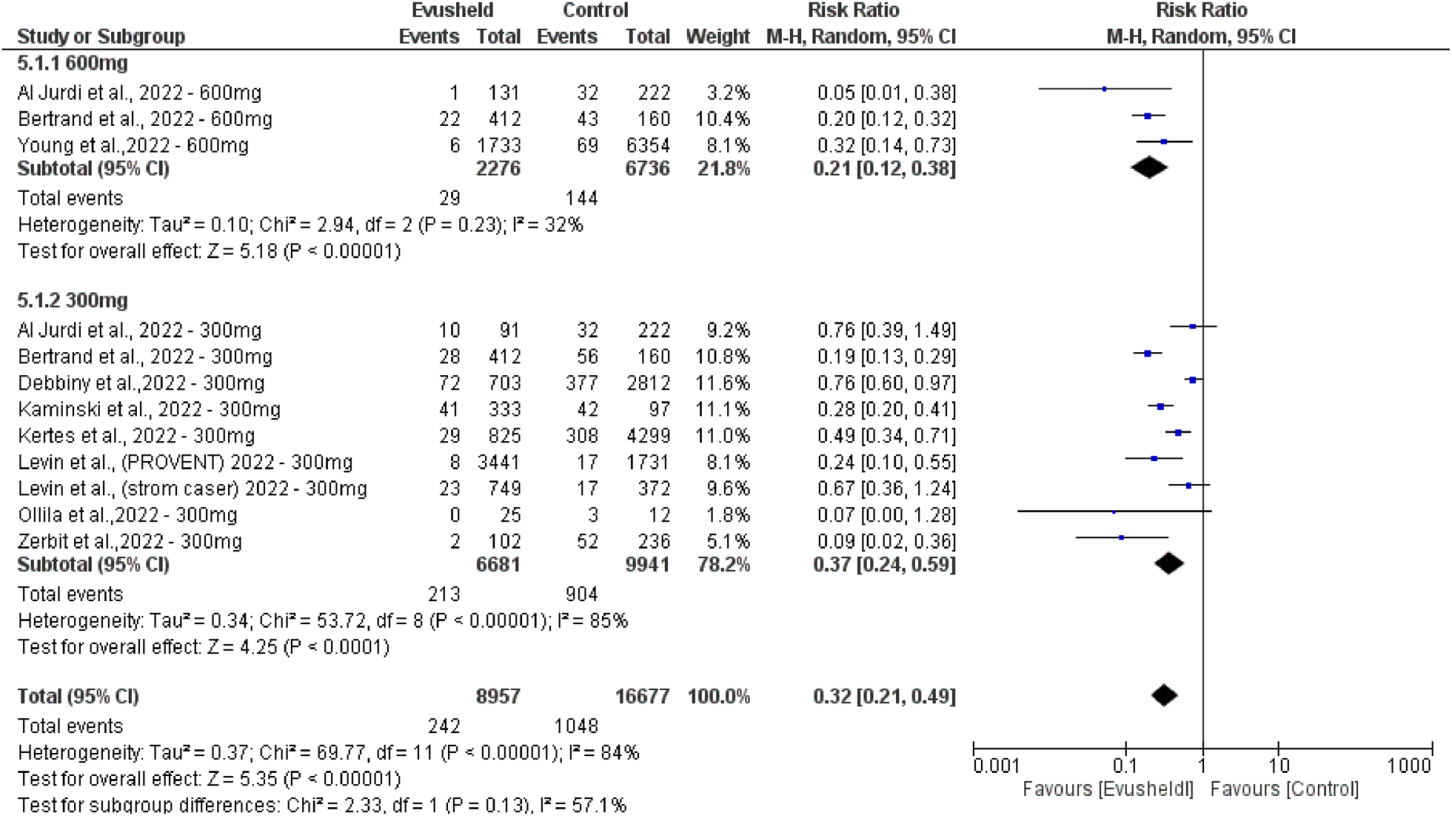
The forest plot reveals the correlation between Evusheld administration as prophylaxis and the rate of COVID-19 infections in double-arm subgroup

Heterogeneity evidence was found between 300 and 600 mg groups (*p* 0.13, *I*^2^ = 57.1%). Low heterogeneity was observed in the 600 mg arm (*p* = 0.23, *I*^2^ = 32%). High heterogeneity was observed in the 300 mg arm (p < 0.00001, *I*^2^ = 85%), and after performing leave-one-test on nine studies, high heterogeneity still existed, as shown in Fig. 4.

Single-arm subgroup analysis of COVID-19 infections by comparing Evusheld 300 mg and 600 mg dose groups included 24 studies. The 300 mg group included 12 studies with 253 infections from 8762 patients. The 600 mg group included 7 studies with 71 infections from the l of 3142 patients.

The results revealed a highly significant difference in the number of COVID-19 infections between the two compared doses (*p* < 0.0001), where COVID-19 infections decreased from (2.887%) in the Evusheld 300 mg group to (2.25%) in the Evusheld 600 mg group.

**Fig. 5:**
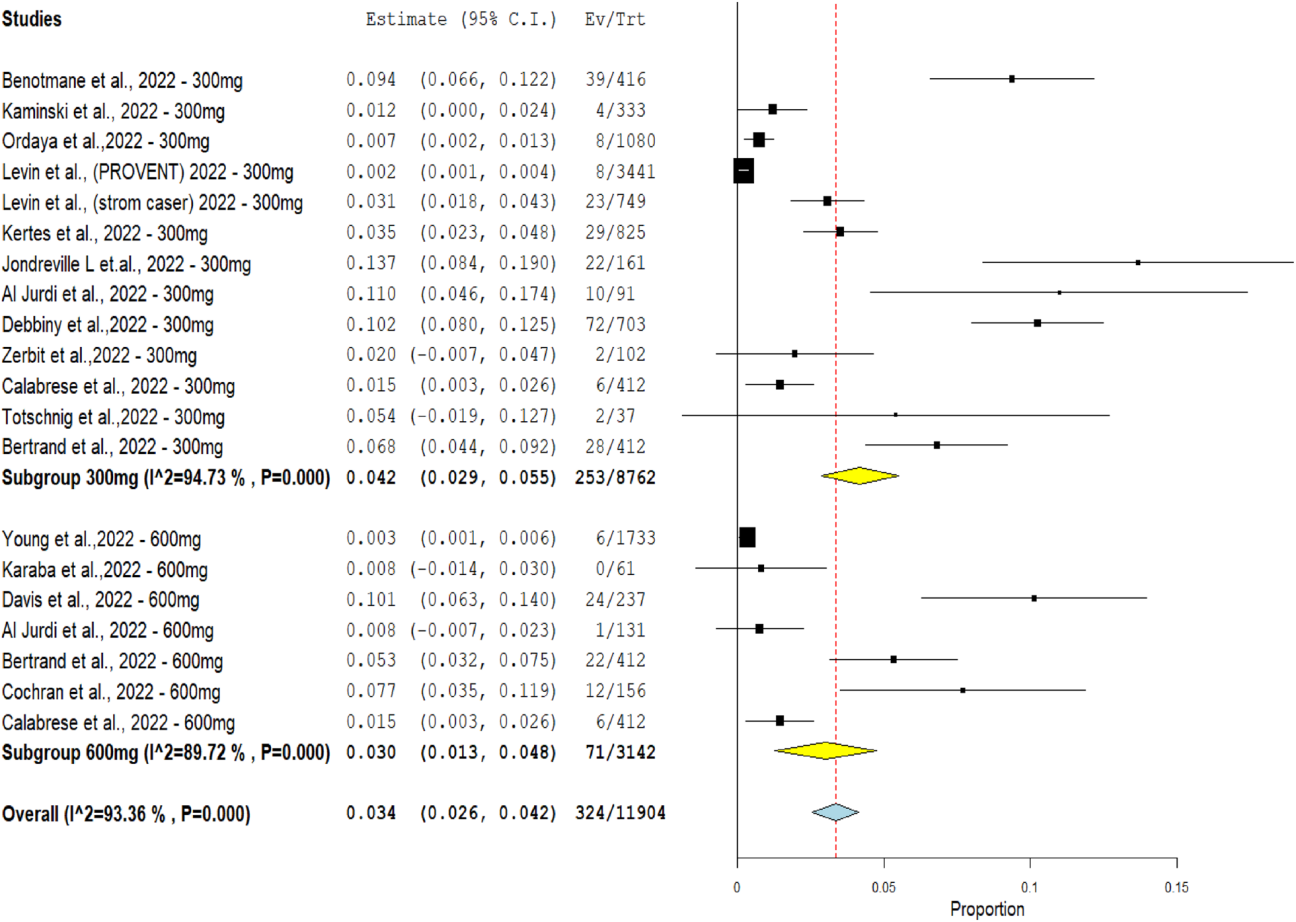
The forest plot demonstrates the association between Evusheld administration as prophylaxis and the rate of COVID-19 infections in the single-arm

High heterogeneity was observed in the 300 mg arm (*p* = 0.000, *I*^2^ = 94.73%), and high heterogeneity was observed in the 600 mg arm (*p* < 0.00001, *I*^2^ = 89.72%), as shown in Fig. 5.

##### Mortality rate

Double-arm subgroup analysis of the mortality rate between Evusheld dose groups 300 mg and 600 mg included 11 studies. The 600 mg dose group included three studies with 2276 patients compared to 6736 patients in the control group. The 300 mg dose group included eight studies with 5891 patients compared to 6903 in the control group.

The results revealed no significant difference in the mortality rate between the two dose groups (*p* = 0.29). However, there was a significant decrease in mortality rate favoring Evusheld 600 mg dose group over the control group (*p* = 0.01). Similarly, there was a highly significant decrease in the mortality rate favoring the Evusheld 300 mg dose group over the control group (*p* < 0.0001). Collectively, there was a highly significant decrease in the mortality rate favored both doses of Evusheld groups over the control groups (*p* < 0.0001).

**Fig. 6:**
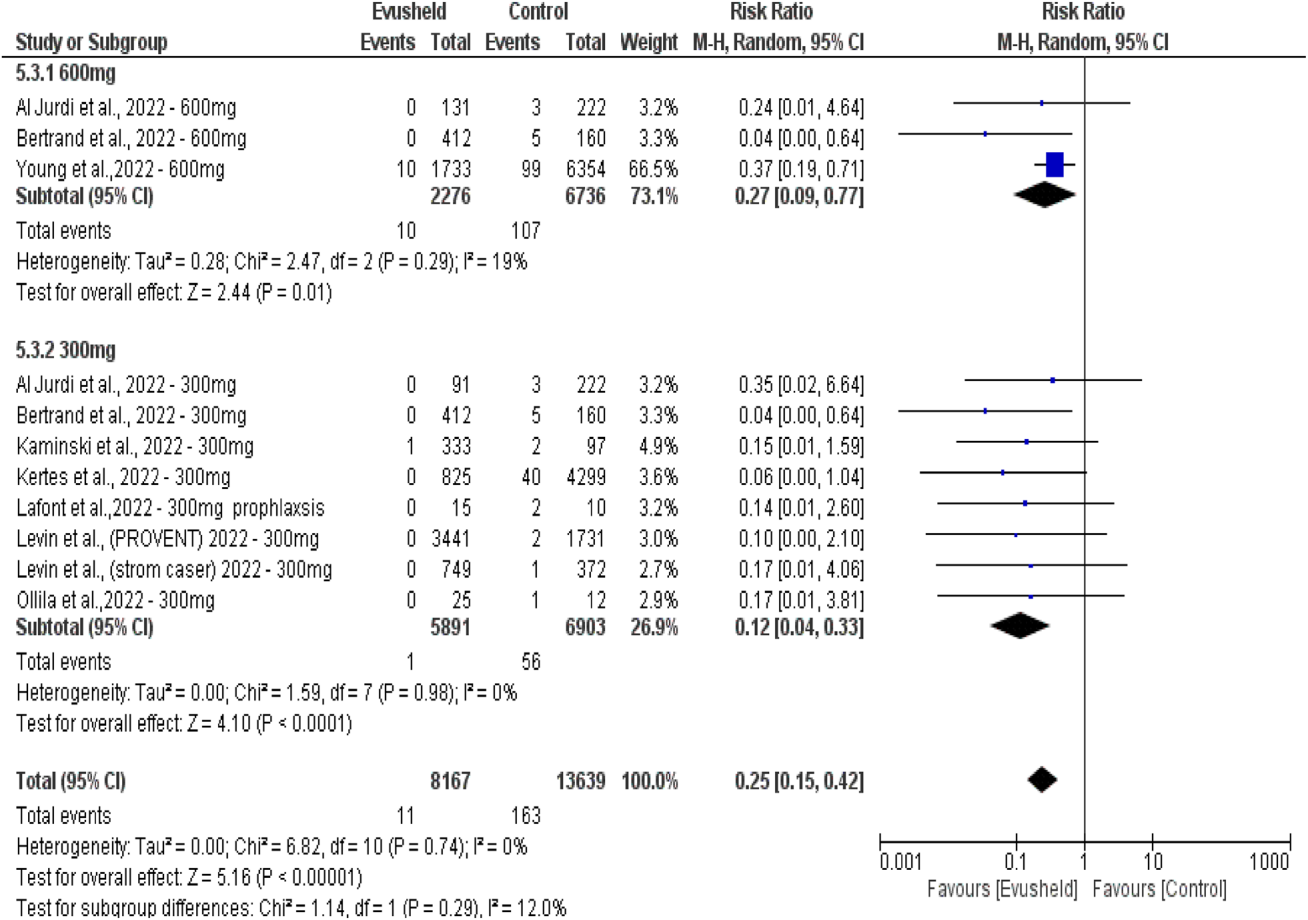
The forest plot shows the correlation between Evusheld administration as prophylaxis and the mortality rate of COVID-19 infections in double-arm subgroup

Low heterogeneity evidence was found between 300 and 600 mg dose groups (*p* = 0.029, *I*^2^ = 12%). Low heterogeneity was observed within the 600 mg dose group (*p* = 0.029, *I*^2^ = 29%). No heterogeneity was observed in the group of 300 mg doses (*p* = 0.98, *I*^2^ = 0%), as shown in Fig. 6.

The single-arm subgroup analysis of the mortality rate by comparing Evusheld dose groups 300 mg and 600 mg included thirteen studies. The 300 mg Evusheld group included nine studies with three deaths from 75 of 08 patients. The 600 mg Evusheld group included four studies with 11 deaths from 2432 patients.

The results showed no substantial difference in the mortality rate among the two compared doses (*p* = 0.228). However, the mortality rate increased from (0.04%) in the Evusheld 300 mg arm to (0.45%) in the Evusheld 600 mg arm.

**Fig. 7:**
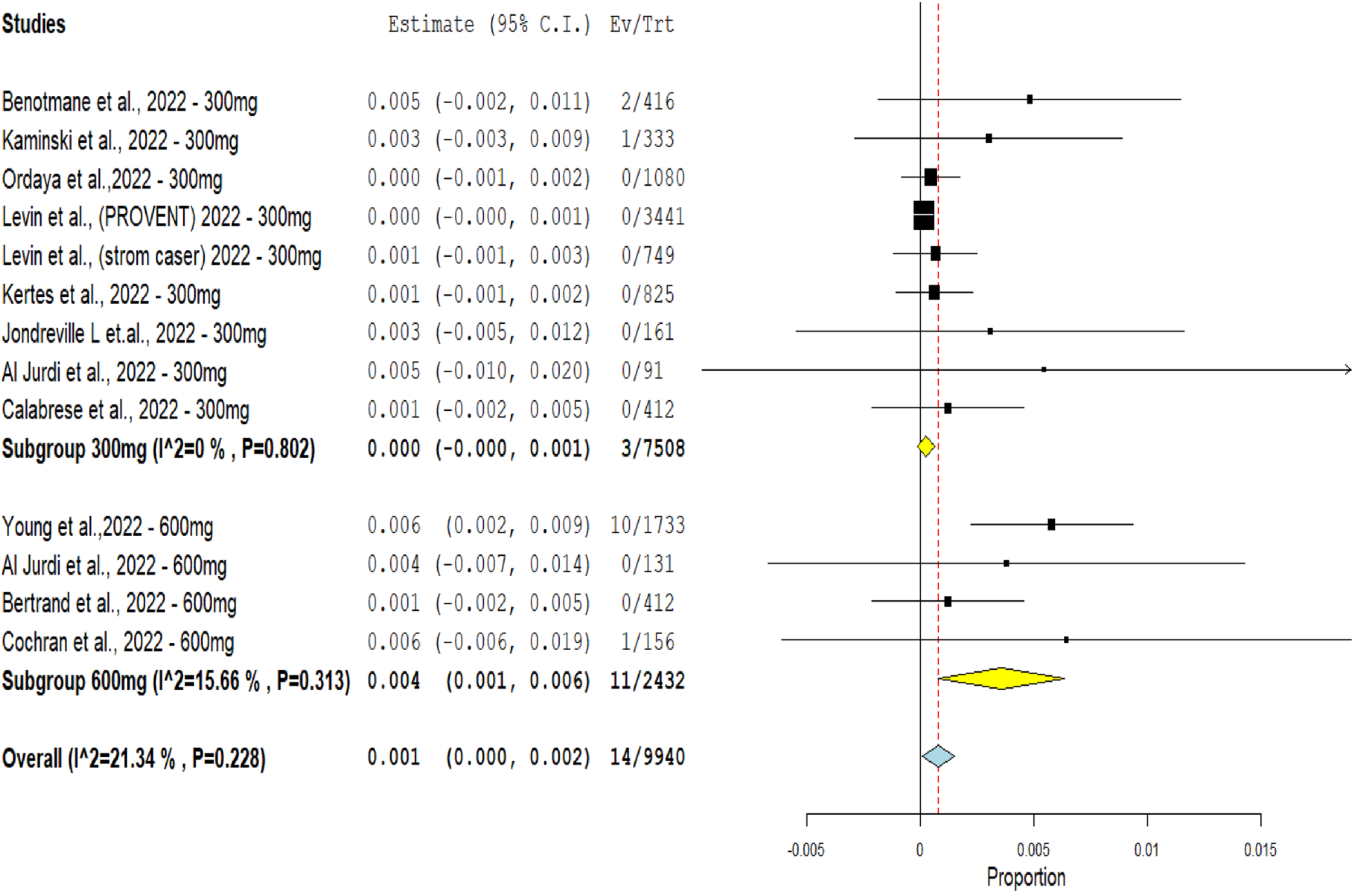
The forest plot reveals the correlation between Evusheld administration as prophylaxis and the mortality rate of COVID-19 infections in single-arm

No heterogeneity was observed in the group of dose 300 mg (*p* = 0.802, *I*^2^ = 0%). Low heterogeneity was observed in the group of dose 600 mg (*p* = 0.313, *I*^2^ = 15.66%), as shown in Fig. 7.

##### Required hospitalization

Double-arm subgroup analysis of the hospitalization rate by comparing Evusheld dose groups 300 mg and 600 mg included nine studies. The 600 mg dose group included three studies with 2276 patients compared to 6736 patients in the control group. The 300mg dose group included six studies with 2376 patients compared to 7600 patients in the control group.

The results revealed no significant difference in patients who required hospitalization between the two compared dose groups (*p* = 0.25). However, there was a significant decrease in patients requiring hospitalization, favoring the Evusheld 600 mg group over the control group (*p* < 0.00001). Additionally, there was a highly significant decrease in patients who required hospitalization, favoring the Evusheld 300 mg group over the control group (p < 0.00001). There was a highly significant decrease in the mortality rate favoring two doses of Evusheld groups than control groups (*p* < 0.00001).

**Fig. 8:**
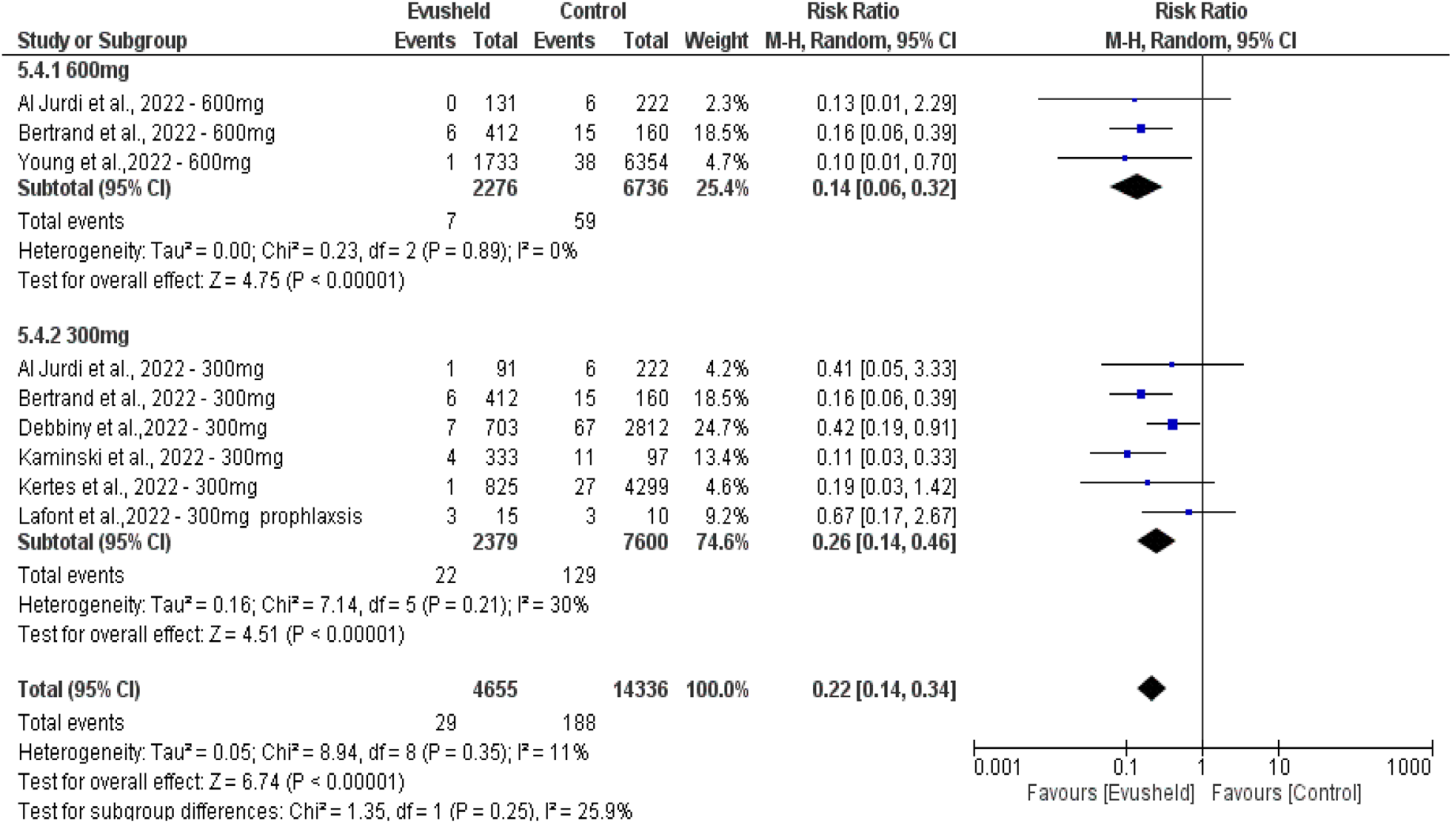
The forest plot shows the association between Evusheld administration as a prophylaxis and infection hospitalization rate of COVID-19 infections in the double-arm group

Heterogeneity evidence was found between 300 and 600 mg dose groups (*p* = 0.25, *I*^2^ = 25.9%). No heterogeneity was observed within the 600 mg arm (*p* = 0.89, *I*^2^ = 0%). Low heterogeneity was observed in the 300mg arm (*p* = 0.21, *I*^2^ = 30%), as shown in Fig. 8.

The single-arm subgroup analysis of the hospitalization rate by comparing the Evusheld dose groups 300mg and 600 mg included 14 studies. The Evusheld 300 mg group included nine studies with 34 patients requiring hospitalization from 4433 patients. The Evusheld 600 mg group included five studies with nine patients requiring hospitalization from 2493 patients.

The results revealed a significant hospitalization rate difference between the two doses (*p* < 0.0001), where the hospitalization rate percent decreased from (0.766%) in the Evusheld 300 mg group to (0.36%) in the Evusheld 600 mg group.

**Fig. 9:**
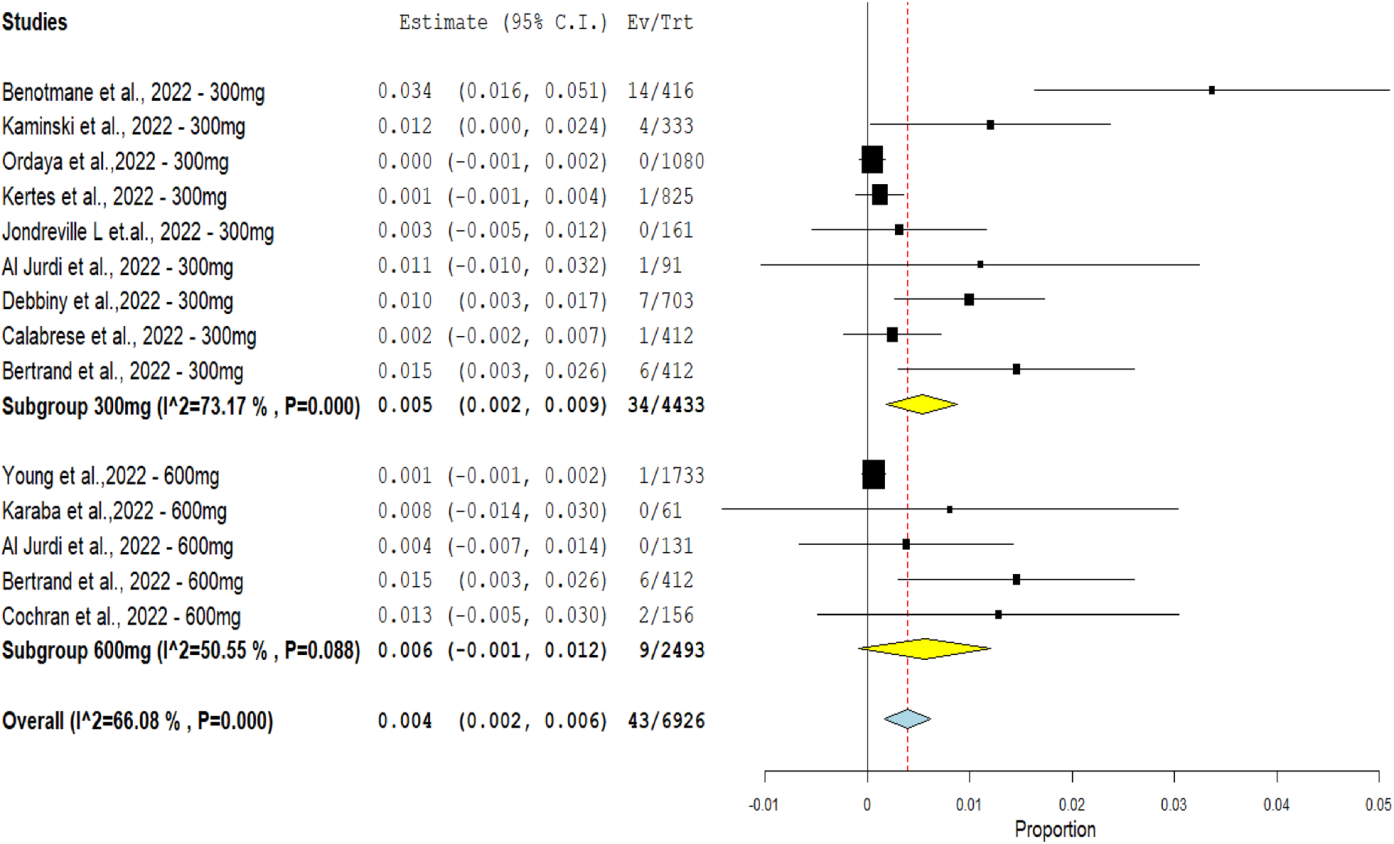
The forest plot shows the relationship between Evusheld administration as prophylaxis and the hospitalization rate of COVID-19 infections in a single-arm subgroup

Heterogeneity was observed in the 300 mg dose group (*p* < 0.0001, *I*^2^ = 73.17%). Low heterogeneity was observed in the 600 mg dose group (*p* = 0.088, *I*^2^ = 50.55%), as shown in Fig. 9.

#### The analysis for the treatment

Analysis of the *mortality rate* within the Evusheld treatment group (includes both 300 mg and 600 mg dose studies) compared to the control group. This analysis included three studies (two studies used the dose of 600 mg, and one used 300 mg) with a total of 1130 patients in the treatment group and 1132 patients in the control group.

**Fig. 10:**
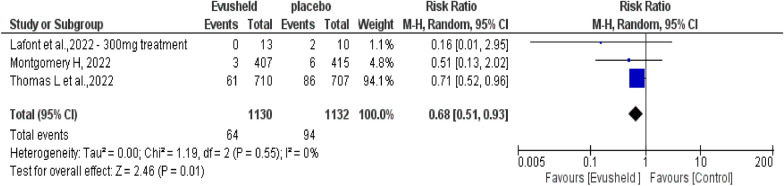
The forest plot reveals the correlation between Evusheld administration as a treatment and the mortality rate of COVID-19 infections

The results revealed a significant decrease in the mortality rate favoring the Evusheld treatment group over the control group (*p* = 0.01). No heterogeneity evidence was found (*p* = 0.55, *I*^2^ = 0%) between the treatment and control groups, as shown in Fig. 10.

## Discussion

The objective of our review is to recognize the favoring effect of the high dose of Evusheld (600 mg) over the lower dose (300 mg) in the prophylaxis against COVID-19 for higher-risk individuals. In addition, we aimed to report the clinical outcomes when Evusheld is used for immunocompromised COVID-19 patients. It included four randomized controlled trials [11, 39, 40, 42], 16 observational cohort studies [27, 29, 31–38, 41, 43–48], and two case series [28, 30], which were involved in the meta-analysis. 8967 patients received Evusheld (2276 received 600mg, 6691 received 300mg), and 16,684 received placebo.

All patients were immunocompromised with different comorbidities, including solid organ transplant, cancer, multiple sclerosis, hypertension, cardiac disease, asthma, and diabetes. Our review was sub-grouped into main categories regarding the dose, Evusheld efficacy as prophylaxis or treatment. Additionally, there is another classification regarding the type of study: double arm or single arm.

### The efficacy of the drug as prophylaxis

The overall result of the meta-analysis found that the difference between the two doses of Evusheld (300 and 600 mg) was insignificant. However, compared to the control group, even during primarily Omicron variant time, there was a highly significant effect of both doses in decreasing the combined rate of COVID-19 infections, mortality, and the need for hospitalization.

The outcome of this meta-analysis was consistent with some published earlier meta-analyses. Soeroto et al. [18] showed that the prophylaxis treatment with Evusheld may reduce the rate of SARS-CoV-2 infection, COVID-19 hospitalization, and mortality when matched with a placebo. Furthermore, another study by Wang et al. demonstrated that the administration of Evusheld to COVID-19 patients has considerable protection against COVID-19 and anti-mortality benefits, with no adverse impacts on developing severe side effects in patients [50]. In addition, Alhumaid et al. [17] discussed in their largest meta-analysis that using the Evusheld in high-risk patients for COVID-19 infection considerably decreased the SARS-CoV-2 infections and was linked to increased side effects. Evusheld performed better than placebo against COVID-19 regarding overall rates of severe and symptomatic COVID-19, its hospitalization, ICU admission, death, COVID-19's Omicron variant neutralization, and oxygen therapy.

However, there are some considerable variations between the previously published analyses and our current systematic review and meta-analysis. Fortunately, our study was the first to analyze the difference between the two doses of Evusheld, including a relatively high number of studies up to 21. In addition, we discussed the clinical outcomes of using Evusheld in prophylaxis and treatment of COVID-19 infection, but the others studied only prophylaxis concerns.

Our single-arm analysis showed a highly significant difference between the two doses, decreasing the number of COVID-19 infections. The dose of 600 mg decreased COVID-19 infections more than the dose of 300 mg. However, the double-arm analysis revealed no considerable difference between these two doses. This difference may be related to the limited research number of double-arm subgroups.

- The difference in mortality rates: (Evusheld 300 mg vs. 600 mg)

Our overall single and double-arm analysis determined no significant variation between the two doses in the mortality rate. However, the mortality rate was higher in the Evusheld 600 mg group (0.45%) than in the Evusheld 300 mg group (0.014%). By comparing each dose to the control, there are highly significant effects of Evusheld 300mg in decreasing the mortality rate compared to the control group. However, there was no significant difference in the higher trials.

- The difference in hospitalization rate (Evusheld 300 mg vs. 600 mg)

Our results of double-arm studies showed no significant difference in patients who required hospitalization between the two doses. However, the single-arm results revealed a significant difference where the dose of 600 mg decreased a higher number of patients who required hospitalization than the dose of 300 mg. This analysis difference may be due to the restricted number of accessible studies.

- The difference in adverse effects of Evusheld (300 mg vs. 600 mg)

Our review defined that the higher dose of Evusheld reported some adverse effects on immunocompromised patients. However, most of the adverse events related to drugs or drugs with other combination medicines were described in solid organ recipients [43, 50], including gastrointestinal disorders (nausea, vomiting, abdominal pain, diarrhea, [6, 36, 43] Nevertheless, there were a few serious adverse effects on the heart, included heart failure, atrial fibrillation, pericarditis, required cardioversion, and mild/moderate cardiac allograft rejection. [11, 36, 42] So, that dose of 300 mg may help reduce the adverse events with the same efficacy as the higher dose.

### The efficacy of the drug as a COVID-19 treatment

Our analysis included three studies: two RCT trials used Evusheld 600mg, and one observational retrospective study used Evusheld 300mg. In those studies, Evusheld was administrated as a treatment in immunocompromised patients who were already infected with COVID-19. The overall results revealed that Evusheld (300 and 600 mg) significantly decreased the mortality rate than the placebo.

Although this review may have found that comparing the two doses of Evusheld is insignificant, 600 mg should be further studied as it appears to favor the effect over 300 mg for COVID-19 prophylaxis and treatment.

In this study, two dosages of Evusheld (300 mg and 600 mg) are assessed for their preventive efficacy in immunocompromised patients. Overall, there is no discernible difference in the preventive efficacy between the doses; nevertheless, in line with previous meta-analyses, both show a significant decrease in COVID-19 infections, hospitalizations, and mortality compared to the control group.

In single-arm analysis, the new dose comparison shows a significant benefit for the 600 mg dose; however, in double-arm analysis, the small number of studies yielded equivocal results. Overall mortality rates are similar, yet there is cause for concern given the higher rate in the 600 mg group. The dual analytical method of the study is its strongest point. The greater dose's side effects, particularly in recipients of solid organs, highlight the necessity of a balanced risk–benefit analysis. Furthermore, as COVID-19 treatments in immunocompromised patients, both doses effectively lower mortality rates. The research offers complex insights into the relative benefits, side effects, and therapeutic potential of Evusheld in this susceptible population.

The study's strengths include its novel approach to methodically examining dose differences, clinical significance to high-risk populations, different study types with a large sample size, and its unique focus on comparing two Evusheld doses in immunocompromised persons. The thorough methodology, which considers the Omicron variation and uses single-arm and double-arm analyses, increases the study’s depth. Furthermore, it provides important safety data regarding the increased dosage, supporting clinicians’ and legislators’ well-informed decision-making.

However, this study had some limitations. First, there was Heterogeneity between the studies, as they included a large number of retrospective cohort studies and case series. We found only three randomized controlled trials. Additionally, only three studies of both two doses were involved in the treatment meta-analysis owing to the restricted number of studies using Evusheld as a treatment. Also, we did not identify and compare the safety profile of each dose of Evusheld. Finally, as we only considered English-language studies, our findings' generalizability is constrained.

The study's suggestions for clinical practice emphasize the necessity for a balanced approach and stress the significance of giving Evusheld (300 mg and 600 mg) to immunocompromised individuals with careful dose evaluation. Physicians should perform comprehensive risk–benefit analyses, accounting for the greater dose’s possible prophylactic efficacy, side effects, and death rates. The results validate the therapeutic efficacy of Evusheld as a COVID-19 treatment in immunocompromised persons, hence recommending its use in managing cases in this high-risk population.

Future research should focus on fine-tuning Evusheld's dosage schedules by thoroughly examining dose-dependent effects, focusing on large-scale randomized controlled studies to provide strong proof. It is imperative to prioritize establishing thorough, long-term safety profiles, particularly in certain patient groups. Studies comparing the efficacy of other COVID-19 therapies to the current one will yield important information for optimizing treatment. Furthermore, it is advised to perform subgroup studies based on certain comorbidities within the immunocompromised population to customize recommendations for various patient groups and acknowledge variations in the risk–benefit profile among people with various medical problems.

## Conclusion

This study thoroughly evaluates the effectiveness of Evusheld in treating and preventing COVID-19 in immunocompromised patients, focusing on two dosages (300 mg and 600 mg). Even during the Omicron version, both doses dramatically lower COVID-19 infections, mortality, and hospitalization compared to a control group. In single-arm analyses, however, the 600 mg dose shows a stronger preventive effect along with more side effects. A thorough risk–benefit analysis is essential. In comparison to a placebo, Evusheld significantly lowers the death rates of immunocompromised COVID-19 patients, demonstrating the treatment's promise. Overall, the study highlights the necessity for careful dosage considerations in this high-risk population and offers nuanced insights into the effects of Evusheld. The study's recommendations for clinical practice highlight the value of cautious dosing, patient-specific considerations, and customized decision-making. Future studies should focus on improving our knowledge of the best way to utilize Evusheld, resolving existing issues, and adding evidence-supporting guidelines for managing COVID-19 in immunocompromised patients.

### Supplementary Information

**Additional file 1. **Search strategy

**Additional file 2: Table S1.** This table shows the Newcastle–Ottawa quality assessment of 16 observational cohort studies. **Table S2.** Quality Assessment Tool for Case Series Studies.

## Data Availability

All data are available to the public.

## Abbreviations

Evusheld: Tixagevimab/cilgavimab
mAbs: Monoclonal antibodies
I.M.: Intramuscular
PrEP: Pre-exposure prophylaxis
WHO: World Health Organization
FDA: Food and Drug Administration
SOC: Standard of care
RCTs: Randomized controlled trials
