# Supplementary material for: The efficacy of tixagevimab/cilgavimab (Evusheld) in prophylaxis and treatment of COVID-19 in immunocompromised patients: a systematic review and meta-analysis
Source: Eur J Med Res. 2024 Jan 5;29:27. doi: 10.1186/s40001-023-01549-x (PMC10768288; doi:10.1186/s40001-023-01549-x)
Supplement: Supplementary file 1 — Additional file 1. Search strategy [file 40001_2023_1549_MOESM1_ESM.docx]

**Search strategy**

((Tixagevimab OR" INN-tixagevimab "AND (Cilgavimab) OR ("Tixagevimab cilgavimab ") OR Evusheld OR" neutralising monoclonal antibody combination" OR “SARS-CoV-2 neutralizing antibody” OR "cilgavimab and tixagevimab drug combination")

AND (COVID-19 OR COVID19 OR COVID 19 OR Coronavirus OR 2019-nCoV OR "Severe Acute Respiratory Syndrome Coronavirus 2" OR "SARS-COV-2" OR "SARS CoV 2" OR NCOV OR "sars-cov-2)
