# Supplementary material for: The efficacy of tixagevimab/cilgavimab (Evusheld) in prophylaxis and treatment of COVID-19 in immunocompromised patients: a systematic review and meta-analysis
Source: Eur J Med Res. 2024 Jan 5;29:27. doi: 10.1186/s40001-023-01549-x (PMC10768288; doi:10.1186/s40001-023-01549-x)
Supplement: Supplementary file 2 — Additional file 2: Table S1. This table shows the Newcastle–Ottawa quality assessment of 16 observational cohort studies. Table S2. Quality Assessment Tool for Case Series Studies. [file 40001_2023_1549_MOESM2_ESM.docx]

**Table S1.** This table shows the Newcastle-Ottawa quality assessment of 16 observational cohort studies.

| **The first author, the Year** | **Selection** | | | | **Comparability** | **Outcome** | | | **Total Score** |
| --- | --- | --- | --- | --- | --- | --- | --- | --- | --- |
|  | **Representativeness of the exposed cohort** | **Selection of the non-exposed cohort** | **Ascertainment of exposure^1^** | **The outcome did not occur at the start of the study^2^** | **Control for 2 important factors^3,4^** | **Assessment of outcome** | **Follow-up long enough** | **Adequacy of follow-up of cohort^5^** |  |
| Al Jurdi et al., 2022 (44) |  | * | * | * | ** | * | * | * | 8 |
| Aqeel et al., 2022 (49) |  | * | * | * | * | * |  | * | 6 |
| Benotmane et al., 2022 (39) |  | * | * | * | * | * |  | * | 6 |
| Bertrand et al.,  2022 (45) |  | * | * | * | * | * | * | * | 7 |
| Calabrese et al.,  2022 (38) |  | * | * | * | ** | * | * | * | 8 |
| Cochran et al.,  2022 (37) |  | * | * | * | ** | * | * | * | 8 |
| Davis et al.,  2022 (36) |  | * | * | * | ** | * | * | * | 8 |
| Kaminski et al.,  2022 (46) |  | * | * | * | * | * | * | * | 7 |
| Karaba et al.,  2022 (47) |  |  | * | * | ** | * |  | * | 6 |
| Debbiny et al.,  2022 (35) |  | * | * | * | ** | * | * | * | 8 |
| Nguyen et al.,  2022 (28) |  | * | * | * | ** | * |  | * | 7 |
| Ollila et al.,  2022 (30) |  | * | * | * | * | * |  | * | 6 |
| Totschnig et al.,  2022 (34) |  | * | * | * | ** | * | * | * | 8 |
| Young et al.,  2022 (32) |  | * | * | * | ** | * | * | * | 8 |
| Zerbit et al.,  2022 (33) |  | * | * | * | ** | * | * | * | 8 |
| Jondreville L et.al., 2022 (42) |  | * | * | * | * | * |  | * | 6 |
| Kertes et al.,  2022 (48) |  | * | * | * | * | * |  | * | 6 |

**Table S2.** Quality Assessment Tool for Case Series Studies.

| **First author, Year** | **Study Design** | **Total score** | **Quality Assessment of Study** |
| --- | --- | --- | --- |
| Goulenok et al., 2022 (50) | Retrospective  case-series | 8 | Good |
| Lafont et al.,2022 (29) | Retrospective  case-series | 8 | Good |
| Ordaya et al., 2022 (31 ) | Retrospective  case-series | 8 | Good |
